# Supplementary material for: A tool to evaluate the impact of lived experience involvement in research: the Brain and Genomics Hub Impact Log protocol
Source: Wellcome Open Res. 2026 Oct 14;11:562. Originally published 2026 Aug 28. [Version 2] doi: 10.12688/wellcomeopenres.27426.2 (PMC13694935; doi:10.12688/wellcomeopenres.27426.2)
Supplement: Peer review history

# Reviewer report: Reviewer response for version 2

**DOI:** 10.21956/wellcomeopenres.30793.r175603

**Published:** 2026-10-23

**Authors:** Emilie Hudson

**Affiliations:** 1 Université de Montréal, Montréal, Canada

I was thrilled to read this LE-led, co-produced protocol for the design and dissemination of the Impact Log. As someone conducting LE-led research, I found the tool useful and practical, and I hope it is widely adopted across a variety of projects.

**Strengths**

- The need for this work is clearly justified and should be compelling to researchers outside LE research paradigms or newer to LEI in their projects.
- The inclusion of researcher positionality and transparency is very thoughtful. The authors’ structure for author disclosures is easily replicable by other researchers.
- The literature review of 96 documents is extensive.
- The authors took the first round of reviewer comments into account, clarifying and expanding where appropriate. The forms are now in the main text, and the extended materials were available, so the development of the Impact Log and how to use it are very clear.
- The analytic process is well detailed. I was glad to see that LE advisors will be included in analysis, and I am curious to see the framework they develop.
- The limitations (funding, lack of LE leadership, health challenges, difficulty maintaining anonymity) are well spelled out.

**Points for the authors to consider**

1. *Protocol and results.* The article is a protocol but also includes completed work, such as the literature review, the preliminary feedback from the eight people with LE, and the LE perspectives results (Table 7). A sentence or two could make clear that these are preliminary works informing the protocol, so readers know what is planned and what is done.
2. *Specificity to the Brain and Genomics Hub.* The Hub combines a recruited cohort (B-SPRINT) with a linked-records e-Cohort, and researchers on such types of projects may not routinely consider LEI. The LE perspectives results and Table 7 are fairly generic about the benefits of LEI; more context on how the Impact Log applies to these designs would help. However, I do understand the wish for broad adaptability, and this reflection may come with the study itself.
3. *Terminology and roles.* There are many differing terms in the LE research and LEI space to describe roles and positions. I appreciated the paragraph on “dual expertise”. However, where members hold clinical or professional expertise alongside research and lived experience, “dual” may be confusing. Could the authors expand on how those with “dual” expertise (Specialists) differ practically, including in terms of power within the research group, and how their forms differ from the LEI form and the researcher form?
4. *Less-resourced settings.* The remunerated-activities details on page 8 are interesting and indicate a context with funding allocated for LEI and strong beliefs in its importance. Do the authors have suggestions for successful implementation in less-resourced settings? The paper has two dissemination paragraphs and a “Please keep in touch” section about adoption elsewhere, so brief guidance would be welcome.

Is the study design appropriate for the research question?

Yes

Is the rationale for, and objectives of, the study clearly described?

Yes

Are sufficient details of the methods provided to allow replication by others?

Yes

Are the datasets clearly presented in a useable and accessible format?

Yes

Reviewer Expertise:

Lived experience research, mental health services, mental health crisis, police response, mental health nursing

I confirm that I have read this submission and believe that I have an appropriate level of expertise to confirm that it is of an acceptable scientific standard.

# Reviewer report: Reviewer response for version 2

**DOI:** 10.21956/wellcomeopenres.30793.r174010

**Published:** 2026-10-23

**Authors:** Martina Odette McGrath

**Affiliations:** 1 The University of Melbourne, Parkville, Australia

Firstly, I wish to thank the authoring team for producing such a timely protocol. I also want to thank them for taking great care to incorporate our reviewer suggested improvements. 

 Beyond, this I have no further comments to make.

Is the study design appropriate for the research question?

Partly

Is the rationale for, and objectives of, the study clearly described?

Yes

Are sufficient details of the methods provided to allow replication by others?

Partly

Are the datasets clearly presented in a useable and accessible format?

Partly

Reviewer Expertise:

lived experience workforce leadership and development, social and health equity in suicide prevention and mental health

I confirm that I have read this submission and believe that I have an appropriate level of expertise to confirm that it is of an acceptable scientific standard.

# Reviewer report: Reviewer response for version 1

**DOI:** 10.21956/wellcomeopenres.30188.r166554

**Published:** 2026-09-17

**Authors:** Martina Odette McGrath

**Affiliations:** 1 The University of Melbourne, Parkville, Australia

**Recommendation:** **Approved with Reservations**

 Thank you for the opportunity to review this study protocol. I found this to be an interesting, timely and potentially very useful piece of work. The development of practical methods for documenting and evaluating the impact of lived experience involvement (LEI) is an important gap in research, and I particularly welcomed the strong lived experience leadership and co-production evident throughout this project.

 I also appreciated the exploratory nature of the Impact Log and noting its evolution will be iterative and ongoing. The authors appropriately acknowledge that this is a novel tool that does not incorporate established outcome measures and propose iterative evaluation and refinement during implementation. Overall, I am supportive of the manuscript, and my comments are primarily intended to improve clarity and strengthen the protocol.

**Minor revision points:**

**1. The Impact Log itself and access to the Extended Materials**

 My main difficulty in reviewing the manuscript was surprisingly fundamental: I was unable to access the Impact Log forms themselves.

 The stated primary aim of the protocol is to present a novel co-produced tool, the Impact Log, for evaluating the impact of LEI. The manuscript states that the three Impact Log forms are provided in Extended Materials 1–3; however, these materials were not available to me through the reviewer portal. While the manuscript provides a useful description of the forms and their principal questions, this doesn’t enable me to asses of the wording, structure, response options, accessibility, burden or relationship between the three versions.

 This is particularly important because subsequent aspects of the protocol—including the proposed quantitative and qualitative analyses—depend upon the content and structure of these instruments.

 Could the journal editors/authors please ensure that Extended Materials 1–3 is available to reviewers and readers. My assessment of the Impact Log itself is necessarily limited without access to these materials.

**2. Clarify precisely what constitutes the “Impact Log”**

 Could the authors please be consistent with their use terminology describing the intervention/tool. Across the manuscript, the terms *Impact Log*, *Impact Log tool*, *Impact Log forms*, *Impact Log framework*, and *Impact Log and LEI framework* appear to be used in overlapping ways.

 At some points, the Impact Log appears to refer to the three data-collection forms. Elsewhere, the “Impact Log framework” seems to encompass a broader methodology or process involving administration, tracking, analysis, feedback and iterative development. The manuscript also describes the Impact Log as embedded within a broader LEI framework. For example, the Aims section moves between the “Impact Log tool,” the wider LEI framework in which it is embedded, and the “Impact Log framework.”

 Could the authors please clearly define these components and terms earlier in the manuscript and then using this same terminology consistently throughout.

**3. Claims regarding replicability and wider adaptation**

 The manuscript makes several statements regarding the Impact Log being replicable and readily adaptable to other settings. I agree that wider adaptation is an important and plausible aim; however, some of the language may currently be slightly stronger than the current evidence supports.

 The authors do appropriately emphasise that this remains a novel tool whose design and analysis will undergo iterative development during implementation. At this stage, then, statements that the Impact Log “provides a replicable tool, easily adaptable for use elsewhere” could be better framed in terms of its **intended adaptability** or **potential for replication and adaptation**, pending implementation and evaluation in other contexts.

**Overall assessment**

 I enjoyed reading this protocol and am strongly supportive of the work, its premise and underlying aims. The lived experience leadership and co-production are substantive rather than peripheral, and the authors demonstrate thoughtful engagement with many of the persistent challenges surrounding LEI, including power, tokenism, diversity, accessibility and sustainability.

 My main concern is not with the overall approach but with the current difficulty in determining precisely what constitutes the Impact Log and, importantly, being able to see and assess the actual tool being presented. Addressing this, alongside greater consistency of terminology and the relatively minor methodological clarifications above, would considerably strengthen what I think is a promising and valuable contribution.

Is the study design appropriate for the research question?

Partly

Is the rationale for, and objectives of, the study clearly described?

Yes

Are sufficient details of the methods provided to allow replication by others?

Partly

Are the datasets clearly presented in a useable and accessible format?

Partly

Reviewer Expertise:

lived experience researcher in public health, primarily in suicide prevention and mental health

I confirm that I have read this submission and believe that I have an appropriate level of expertise to confirm that it is of an acceptable scientific standard, however I have significant reservations, as outlined above.

# Response

**Published:** 2026-09-30

**Authors:** Tania Gergel

**General Response: ** Many thanks for this extremely helpful review and for your support for our project.  We have done our best to integrate all your suggestions and to make the Impact Log Forms much more easily accessible for readers – we hope that this will give you everything you need for a more complete review. We have responded to your comments individually below and all changes have been incorporated into the revised manuscript as tracked changes.

**Response to Minor Revision Point 1 ** We are very sorry that you were unable to view the forms as part of your initial review process and thank you very much for pointing out a lack of clarity/difficulty in locating the forms.  In the initial version, we included the Impact Log forms within the Open Access downloadable Extended Materials file and indicated that they are available as Sections 1-3 of the Extended Materials in the " *Form development*" section, with links to Extended Materials provided in the **Extended Data **Section at the end of the paper. However, we recognise that a) this may be insufficiently clear and b) given the centrality of the Forms to the protocol and our aims to reach a wide readership, it may be better if we include the forms as Tables within the main body of the text.  The editorial team at Wellcome Open have agreed that, for ease and given their centrality, they are in agreement about presenting the Forms as tables within the main text (as well as within the Extended Materials).  We have therefore added the Forms as Tables 4-6 in the main text, while also retaining a formatted and downloadable Word version in the Extended Materials. 

**Response to Minor Revision Point 2**.  Many thanks for pointing this out and for suggesting further clarification and consistency in the terminology we use to indicate the *Impact Log *tool and its surrounding LEI framework within the Brain and Genomics Hub.   We have been through the manuscript and, from the outset, now rephrased relevant sections with tracked changes, so that there is a much clearer demarcation between the *Impact Log *tool and data itself and the wider LEI framework within which it has been designed and will be implemented within the Hub.  We hope that this makes things much clearer for our readers.

**Response to Minor Revision Point 3 ** many thanks for highlighting this – we have adapted this sentence to read “The *Impact Log* and LEI framework also provides a replicable tool, with easy potential adaptability for implementation and evaluation elsewhere in medical research”, and hope that this answers your concerns.

# Reviewer report: Reviewer response for version 1

**DOI:** 10.21956/wellcomeopenres.30188.r166549

**Published:** 2026-09-16

**Authors:** Lavanya Vijayasingham

**Affiliations:** 1 HealwithZeal Global CIC, London, UK

Thank you for the opportunity to review this protocol. I found this an important and timely piece of work. The prospective Impact Log is a useful contribution to an area in which lived-experience engagement is increasingly expected, but where approaches to documenting its contribution, influence and impact remain inconsistent.

 I am broadly supportive of the protocol and have a few minor editorial suggestions that I think would strengthen its conceptual and methodological framing and extend its relevance beyond brain and mental health.

**1. Clarify the gap and link this more directly to the study objective**

 I suggest sharpening the gap-to-objective pathway. Despite growing recognition of lived experience as expertise, there remains no widely accepted gold standard for identifying and evaluating meaningful lived-experience engagement, involvement and, importantly, leadership across global health research. This gap extends beyond brain and mental health and gives this work potentially much wider relevance.

 The authors could articulate this more directly, for example: “In the absence of an established gold standard for identifying and evaluating meaningful lived-experience engagement, involvement and leadership across global health research, this study aims to prospectively apply an Impact Log to understand how lived-experience expertise contributes to and influences research processes and outcomes.” This would make both the need for the study and its potential contribution immediately clearer.

**2. Strengthen the epistemological and methodological positioning**

 The protocol provides useful operational detail, but I would encourage the authors to more clearly describe the epistemological and methodological foundations of the work. For example, is the study situated within social constructionism, recognising accounts of involvement and impact as situated and co-constructed? Does it draw on realist evaluation, particularly an interest in understanding what works, for whom, in what circumstances and through what mechanisms? Or might it be more readily recognisable to researchers as a participatory research or longitudinal case-study methodology?

 Greater clarity about ontology, epistemology and methodology would help readers understand what constitutes knowledge and evidence within the Impact Log, strengthen reproducibility, and clarify what claims can ultimately be made about “impact”.

 Relatedly, I suggest distinguishing more clearly between experience of engagement, contribution, influence, change and impact. Feeling heard is important but is not necessarily evidence that a contribution influenced a decision or changed the research. Charting the course and influence of lived experience voices (and indeed whose lived experience voices) within the process from design to dissemination is important.  

**3. Make reflexivity, positionality and document contribution more explicit in approach**

 I particularly liked Table 1 and think the authors could make more methodological use of it. Rather than simply describing who is in the team, it could explicitly identify the different forms of expertise each team member contributes, for example, lived experience, clinical, methodological, disciplinary or implementation expertise, and also positionality of how their background histories and other aspects of identity contribute to their research or practice lens. This would strengthen reflexivity and positionality (again, commonly under-reported in mainstream research) by making visible the different knowledge assets within the team and acknowledging how these positions may shape what is asked, valued and interpreted.

 There is an interesting parallel here with the CRediT taxonomy in journal publishing, which makes different forms of scholarly contribution visible rather than relying on authorship position as a proxy. A similar principle could be valuable here: who contributed what, at which stage of the research process, and with what consequence? This could also provide a useful mechanism for tracking contributions longitudinally.

**4. Consider power and the ability to influence and lead; and integrate domains into a logic model or theory of change framework**

 For me, an important opportunity is to move beyond measuring the presence or experience of lived-experience contributors towards understanding their power to influence research decisions and processes.

 Meaningful engagement should arguably distinguish between being invited to participate, having an opportunity to contribute, influencing a decision, sharing decision-making power, and having opportunities to initiate or lead work.

 This would align with emerging recommendations on meaningful engagement that move beyond representation towards examining whose priorities shape agendas and resources, whose recommendations are adopted, and whether engagement results in changes to decisions, processes or systems. 

 The authors might therefore consider capturing whether lived-experience recommendations were adopted fully, partially or not at all; whether contributors had influence over study design and process decisions; and whether they had opportunities to lead rather than only respond to researcher-defined agendas.

 The Impact Log could therefore consider capturing a progression such as presence → participation → contribution → influence → shared decision-making → leadership. Or: LE contribution → consideration → decision → adoption/non-adoption → change in research process or product → downstream consequence. Indeed, a logic model or theory of change framework can be developed to guide the implementation and evaluation, which will be very valuable to guide future adoption beyond brain and mental health.

**5. Negative experience, barriers and instances where lived experience did not influence decisions/outcomes are also important**

 Importantly, I would also suggest capturing negative instances and diving deep into where lived-experience contributions do not result in change to better understand how barriers manifest in real-time and across different settings. Understanding why a recommendation was not adopted, whether because of methodological, regulatory, resource, institutional or other constraints, may be as informative as documenting successful influence. It would provide insight into the conditions under which lived-experience expertise can and cannot influence research. 

 Overall, I found this a thoughtful and important protocol that identifies and aims to address a significant gap in research, knowledge and practice. Ultimately, the most interesting contribution may be the opportunity to move beyond asking whether people with lived experience were present or felt heard, towards understanding whether, how and under what conditions their expertise and voice had the power to influence what was thought, decided and done.

 My suggestions are intended primarily to strengthen the conceptual and methodological architecture around the Impact Log and broaden its potential relevance across global health.

Is the study design appropriate for the research question?

Yes

Is the rationale for, and objectives of, the study clearly described?

Yes

Are sufficient details of the methods provided to allow replication by others?

Yes

Are the datasets clearly presented in a useable and accessible format?

Yes

Reviewer Expertise:

Global health, lived experience qualitative research, gender, chronic illness, meaningful engagement, research reflexivity

I confirm that I have read this submission and believe that I have an appropriate level of expertise to confirm that it is of an acceptable scientific standard.

## References

[1] Key advocacy messages for meaningful engagement of people living with NCDs, mental health and neurological conditions. *https://www.knowledge-action-portal.com/en/content/key-advocacy-messages-meaningful-engagement-people-living-ncds-mental-health-and* .

[2] PLOS Global Public Health, charting a new path towards equity, diversity and inclusion in global health. *https://journals.plos.org/globalpublichealth/article?id* .10.1371/journal.pgph.0000038PMC1002127736962122

# Response

**Published:** 2026-09-30

**Authors:** Tania Gergel

**Overall response:** Many thanks for your support for our research and for your extremely helpful suggestions. We have responded to your comments individually below and all changes have been incorporated into the revised manuscript as tracked changes.

**Response to Point 1: **Many thanks for this valuable suggestion about giving clearer indication about a) the motivations; b) wider relevance beyond the mental health context.  We have added an adapted version of your suggested sentence to the penultimate paragraph of Section 1, and have also added the phrase “, both within mental health and other areas of research,” in Para 4 of 3.1 Strengths and Limitations.

**Response to Point 2: ** Many thanks for asking for further clarification re epistemological stance. We had cut any complex epistemological reflections in the initial draft due to both time and concerns about too much complexity. Also, we were careful not to align ourselves with one particular epistemological/methodological approach, as we draw on multiple approaches and want to remain adaptable. However, we recognise that a more explicit statement about some of the ideas we incorporate would be a useful addition.  We have therefore added the following sentence in the Aims section of 2.1 Methods/Design and hope it gives a succinct but helpful summary of the approach we have taken: “The Impact Log draws on principles from participatory research and realist evaluation, alongside a broadly constructionist understanding of LE expertise. Participatory traditions inform our recognition of experiential, interpersonal and relational dimensions of impact. Realist principles guide our distinction between contribution, influence, change and impact, recognising that LEI may activate mechanisms that shape research processes and outcomes. This blended positioning clarifies what constitutes evidence within the Impact Log while maintaining flexibility and adaptability in how the tool is applied.” Also, to further clarify and highlight our intentions to monitor whether and how suggestions arising from LEI are being implemented, so that we have evidence of impact on design, delivery, systems etc., we have added an additional sentence explaining some of this in the *Data Analysis *Section of 2.1 Methods/Design “It will be considered both in itself and in relation to records of LEI activities, suggested modifications and evidence of whether and how these modifications were adopted, including factors relating to non-adoption, as well as examining and documenting further downstream consequences.”.

**Response to Point 3: ** Many thanks for your enthusiasm about the inclusion of Table 1, which identifies relevant LE / other experiences of individual research team members. We were not able to make tracked changes on the manuscript section pertaining to this Table, as it is not included in the main manuscript, but inserted separately by the editorial team at Wellcome Open.  However, to make these points more explicit, and apparent/easy to find we will be requesting the following changes to the introductory ‘ **Public and patient / lived experience involvement**’ section, indicated in track changes in the para below. **Public and patient / lived experience involvement** Initial development of the Impact Log and LEI framework was by LE researchers and the further development and adaptations described below have been LE-led and co-produced by researchers with LE of bipolar and psychosis. The manuscript preparation has been LE-led and co-produced. This co-production strategy integrates researchers with and without direct LE within an equitable research environment recognising all forms of expertise. We therefore hope to provide an example demonstrating the potential of fully co-produced research, led by researchers with LE, despite the health challenges that we face. To highlight the level of LEI, we have added “Table 1[TG1] : Author disclosed information on mental health LE and professional experience”, which shows specific direct and indirect LE, alongside other relevant personal and professional experience for each author. Details of individual contributions from each author can be found under the Author tab for this article, demonstrating more specifically and extensively how extensively LE expertise and leadership has been embedded throughout this project. Full Further full descriptions of LE involvement in this work can be found throughout this paper and, in fuller detail, in the author contributions and details sections and in the GRIPP2 form in Section 4 of the Extended Materials. . For a table showing LE and other relevant experience and expertise amongst the authorial team please see “Table 1: Author disclosed information on mental health LE and professional experience”. This article is framed within the conventional language and structures of medical research, in order to present a thorough and rigorous explanation of this protocol. We do recognise that this could present a barrier to adaptation of the tools presented below by people with LE who do not have research backgrounds. However, as detailed below, we will be working to provide accessible versions of this work for dissemination amongst non-specialist audiences. The Impact Log itself has been designed to maximize accessibility for users.

  [TG1] We will ask Wellcome Open to hyperlink to Table 1 from this section.

**Response to Point 4:** Thanks for suggesting that we outline more explicitly how we will capture impact on design/delivery, systems and leadership.  As detailed in our response to your Point 2 above, we have included an additional sentence explaining some of this in the *Data Analysis *Section of 2.1 Methods/Design “It will be considered both in itself and in relation to records of LEI activities, suggested modifications and evidence of whether and how these modifications were adopted, including factors relating to non-adoption, as well as examining and documenting further downstream consequences.”. We hope that this clarifies our intention to record and evaluate these impacts and how this will be carried out.  We have not explicitly identified a Theory of Change framework at this point, as we were keen to minimise the use of too much technical terminology and were concerned about adding frameworks without extensive explanation.  However, we will be considering the Impact Log through a broad Theory of Change framework.

**Response to Point 5: ** Many thanks for raising this important point. It is certainly our intention to examine negative instances, non-adoption, and barriers – but it seems that we have not made this sufficiently explicit within the Version 1 manuscript.  We hope that the sentence added to the Data Analysis section detailed in our responses to Points 2 and 4 above now covers this point, and we will certainly make sure these considerations are prioritised within the research.
